# Dual Targeting of Glioblastoma Cells with Bispecific Killer Cell Engagers Directed to EGFR and ErbB2 (HER2) Facilitates Effective Elimination by NKG2D-CAR-Engineered NK Cells

**DOI:** 10.3390/cells13030246

**Published:** 2024-01-28

**Authors:** Anne Kiefer, Maren Prüfer, Jasmin Röder, Jordi Pfeifer Serrahima, Malena Bodden, Ines Kühnel, Pranav Oberoi, Winfried S. Wels

**Affiliations:** 1 Georg-Speyer-Haus, Institute for Tumor Biology and Experimental Therapy, 60596 Frankfurt, Germany; 2 Frankfurt Cancer Institute, Goethe University, 60590 Frankfurt, Germany; 3 German Cancer Consortium (DKTK), Partner Site Frankfurt/Mainz, a Partnership between DKFZ and University Hospital Frankfurt, 60590 Frankfurt, Germany

**Keywords:** natural killer cells, NK-92, NKG2D, chimeric antigen receptor, EGFR, ErbB2, bispecific antibody

## Abstract

NKG2D is an activating receptor of natural killer cells that recognizes stress-induced ligands (NKG2DL) expressed by many tumor cells. Nevertheless, NKG2DL downregulation or shedding can still allow cancer cells to evade immune surveillance. Here, we used lentiviral gene transfer to engineer clinically usable NK-92 cells with a chimeric antigen receptor (NKAR) which contains the extracellular domain of NKG2D for target recognition, or an NKAR, together with the IL-15 superagonist RD-IL15, and combined these effector cells with recombinant NKG2D-interacting bispecific engagers that simultaneously recognize the tumor-associated antigens epidermal growth factor receptor (EGFR) or ErbB2 (HER2). Applied individually, in in vitro cell-killing assays, these NKAB-EGFR and NKAB-ErbB2 antibodies specifically redirected NKAR-NK-92 and NKAR_RD-IL15-NK-92 cells to glioblastoma and other cancer cells with elevated EGFR or ErbB2 levels. However, in mixed glioblastoma cell cultures, used as a model for heterogeneous target antigen expression, NKAR-NK cells only lysed the EGFR- or ErbB2-expressing subpopulations in the presence of one of the NKAB molecules. This was circumvented by applying NKAB-EGFR and NKAB-ErbB2 together, resulting in effective antitumor activity similar to that against glioblastoma cells expressing both target antigens. Our results demonstrate that combining NK cells carrying an activating NKAR receptor with bispecific NKAB antibodies allows for flexible targeting, which can enhance tumor-antigen-specific cytotoxicity and prevent immune escape.

## 1. Introduction

Natural Killer Group 2D (NKG2D) is a C-type lectin-like type II transmembrane protein that serves as an activating receptor for natural killer (NK) cells, CD8^+^ T cells, NKT cells, and subpopulations of CD4^+^ and γδ T cells [1,2,3]. In humans, NKG2D interacts with eight stress-induced cell surface ligands (NKG2DL) that include the MHC class-I related molecules MICA and MICB, as well as the UL16-binding proteins ULBP1–ULBP6 [4,5,6]. Due to their enhanced expression in almost all cancer types, NKG2DL are regarded as universal targets for adoptive immunotherapy with effector lymphocytes endogenously expressing NKG2D or engineered with an NKG2D-based chimeric antigen receptor [7,8]. Autologous NKG2D-CAR T cells have demonstrated an acceptable safety profile in phase I clinical trials in patients with acute myeloid leukemia, myelodysplastic syndromes, or multiple myeloma, with anti-leukemic activity and objective responses observed in some of the treated subjects [9,10,11]. Nevertheless, while permitting subsequent allogeneic hematopoietic stem cell transplantation, such responses to NKG2D-CAR T cell monotherapy themselves were not durable [11]. This may in part be due to the low or absent NKG2DL expression in leukemia-initiating cells [12,13]. Furthermore, cancer cells can escape NKG2D-mediated immune surveillance via the proteolytic shedding of NKG2DL. The resulting soluble ligands compete for NKG2D binding, leading to NKG2D internalization and degradation, as well as the desensitization of effector lymphocytes [14,15,16,17,18].

To address the problem of low or absent NKG2DL expression while still employing the NKG2D receptor for targeted tumor cell-killing, different types of bispecific killer cell engagers were developed, which simultaneously interact with NKG2D and a tumor-associated surface antigen, allowing for NKG2D-positive lymphocytes to attack tumors, independent of NKG2DL recognition [19,20]. For NKG2D binding, such molecules harbor domains derived from natural NKG2D ligands like ULBP2 or MICA [21,22,23,24], or NKG2D-specific single-chain fragment variable (scFv) antibodies or nanobodies [25,26,27,28], which are, in each case, linked to a second binding domain that targets a distinct surface antigen expressed by hematological malignancies or solid tumors. Employing a bispecific antibody (termed NKAB-ErbB2) which binds to NKG2D and the tumor-associated antigen ErbB2 (HER2), we previously demonstrated that such killer cell engagers not only redirect cytotoxic lymphocytes endogenously expressing NKG2D to ErbB2-positive cancer cells, but also markedly enhance the specific cytotoxicity of NK and T cells carrying an NKG2D-based CAR (NKAR) [27].

Here, we functionally enhanced the NKAR construct by including a sequence encoding the IL-15 superagonist RD-IL15, which contains the sushi domain of IL-15Rα and affinity-enhanced IL-15_N72D_, and can support the growth and activity of NK cells in the absence of cross-presentation of IL-15 by IL-15Rα [29,30,31]. NKAR, or NKAR and RD-IL15, were expressed in continuously expanding human NK-92 cells as a clinically relevant model. To enable NKG2DL-independent tumor recognition, NKAR-NK-92 and NKAR_RD-IL15-NK-92 cells were applied in combination with NKAB-ErbB2 or a novel bispecific antibody termed NKAB-EGFR. The latter recognizes epidermal growth factor receptor (EGFR) via an N-terminal scFv antibody domain, linked by an IgG4 Fc region to a second NKG2D-binding scFv antibody fragment at the C-terminus. The specific targeting of NKG2D-positive primary NK and NKAR-engineered NK-92 cells by NKAB-EGFR and NKAB-ErbB2 was investigated in cell-killing experiments with tumor cells expressing EGFR and/or ErbB2, and with mixed glioblastoma cell cultures mimicking the heterogeneous target antigen expression often observed in human tumors.

## 2. Materials and Methods

### 2.1. Cells and Culture Conditions

K562 erythroleukemia cells (ATCC, Manassas, VA, USA) were grown in RPMI 1640 medium (Gibco, Thermo Fisher Scientific, Darmstadt, Germany). Murine GL261 glioblastoma cells [32] (kindly provided by Michael C. Burger, University of Frankfurt), and human T98G glioblastoma, MDA-MB468 breast carcinoma, and HEK293T embryonic kidney cells (all ATCC) were propagated in DMEM medium (Gibco). As supplements, 10% heat-inactivated FBS (Capricorn Scientific, Ebsdorfergrund, Germany), 2 mM L-glutamine, 100 U/mL penicillin and 100 μg/mL streptomycin (all Gibco) were added to the culture media. Expi293F cells (Gibco) were propagated in Expi293 expression medium (Gibco). NK-92 cells [33] (kindly provided by NantKwest, Inc., Culver City, CA, USA) as well as NKAR-NK-92 [27] and NKAR_RD-IL15-NK-92 cells (see below) were cultivated in X-VIVO 10 medium (Lonza, Cologne, Germany) with 5% heat-inactivated human AB plasma (German Red Cross Blood Donation Service Baden-Württemberg-Hessen, Frankfurt, Germany) and 100 IU/mL IL-2 (Proleukin; Novartis Pharma, Nürnberg, Germany). Peripheral blood mononuclear cells (PBMCs) were derived from commercially obtained buffy coats from anonymous blood donors (German Red Cross Blood Donation Service Baden-Württemberg-Hessen) by Ficoll–Hypaque density gradient centrifugation. Primary NK cells (pNK) were isolated from buffy coats using the RosetteSep human NK cell enrichment cocktail (STEMCELL Technologies, Cologne, Germany) according to the manufacturer’s instructions. PBMCs and pNK cells were cultured in X-VIVO 10 medium, supplemented with 5% heat-inactivated human AB plasma, 500 IU/mL IL-2 and 50 ng/mL IL-15 (PeproTech, Thermo Fisher Scientific, Darmstadt, Germany).

### 2.2. NK-92 Cells Expressing an NKG2D-Based CAR and RD-IL15

NKAR-NK-92 cells were described previously [27]. These cells express an NKG2D-based chimeric antigen receptor (NKAR) that encompasses an immunoglobulin heavy-chain signal peptide, the extracellular domain of human NKG2D, a (G_4_S)_2_ linker, a Myc-tag and a modified CD8α hinge region, connected to the transmembrane and intracellular domains of CD3ζ. For the co-expression of NKAR and IL-15 superagonist RD-IL15, which consists of an immunoglobulin heavy-chain signal peptide, the IL-15Rα sushi domain and affinity-optimized IL-15_N72D_ [31], codon-optimized NKAR and RD-IL15 sequences were de novo synthesized (GeneArt, Thermo Fisher Scientific, Darmstadt, Germany), connected via a porcine teschovirus self-cleaving peptide (P2A), and introduced to the lentiviral transfer plasmid pHR’SIN-cPPT-SIEW (pSIEW) [34], upstream of the internal ribosome entry site (IRES) and the enhanced green fluorescent protein (EGFP) sequence of the vector. With the resulting transfer plasmid pS-NKAR_RD-IL15-IEW, VSV-G pseudotyped vector particles were produced and used for the lentiviral transduction of NK-92 cells, as described [35]. EGFP-positive cells were selected with a FACSAria fluorescence-activated cell sorter (BD Biosciences, Heidelberg, Germany), and NKAR surface expression by sorted NKAR_RD-IL15-NK-92 cells was confirmed by flow cytometry with a PE-labeled anti-NKG2D antibody (BAT221; Miltenyi Biotech, Bergisch Gladbach, Germany).

### 2.3. Expression and Activity of RD-IL15

To examine the secretion of RD-IL15, supernatant of NKAR_RD-IL15-NK-92 cells, cultured for 3 days at an initial cell density of 1 × 10^6^ cells/mL, was harvested and analyzed by sandwich ELISA with an IL-15-specific capture antibody (34593; R&D Systems, Wiesbaden, Germany), a biotin-conjugated detection antibody specific for IL-15Rα (R&D Systems), and HRP-conjugated streptavidin (Genscript, Piscataway, NJ, USA). Recombinant His-tagged RD-IL15 was used to generate a standard curve for quantification [31]. Culture supernatants of NKAR-NK-92 and parental NK-92 cells that lack RD-IL15 expression served as controls. To analyze the effect of RD-IL15 on proliferation, NKAR_RD-IL15-NK-92 cells were grown for 7 days in medium with or without the addition of recombinant IL-2. On days 0, 1, 4, and 7, the number of viable cells was determined by trypan blue exclusion. NKAR-NK-92 cells were included for comparison. RD-IL15-induced IL-15 receptor signaling was assessed by the detection of phosphorylated STAT5. NKAR_RD-IL15-NK-92 cells were grown overnight in medium lacking IL-2, whole-cell lysates were prepared, and 30 μg of total proteins per lane were analyzed by SDS-PAGE and immunoblotting with antibodies specific for STAT5 (89/Stat5; BD Biosciences) and phospho-STAT5 (C11C5; Cell Signaling Technology, Leiden, The Netherlands), followed by an HRP-conjugated secondary antibody (Sigma-Aldrich, Taufkirchen, Germany) and chemiluminescent detection. NKAR-NK-92 cells served as a control.

### 2.4. Production of Bispecific NKAB Antibodies

Bispecific killer cell engagers (NKAB antibodies) specifically binding to ErbB2 and NKG2D were previously described [27]. Here, we employed an NKAB-ErbB2 molecule consisting of an ErbB2-specific N-terminal single chain fragment variable (scFv) domain [35], fused via an IgG4 Fc region to an NKG2D-specific C-terminal scFv sequence [36]. To generate the similar NKAB-EGFR molecule, a codon-optimized sequence encompassing an immunoglobulin heavy-chain signal peptide, an scFv fragment derived from EGFR-specific antibody 225 [37], hinge, CH2 and CH3 domains of human IgG4 (UniProtKB P01861; amino acid residues 104-327), a (G_4_S)_2_ linker, and the NKG2D-specific scFv antibody fragment was assembled in silico, de novo synthesized (GeneArt) and inserted into mammalian expression vector pcDNA3, resulting in plasmid pcDNA3-NKAB-EGFR.

Recombinant NKAB-EGFR and NKAB-ErbB2 molecules were expressed as secreted proteins in Expi293F cells transiently transfected with the ExpiFectamine 293 transfection kit according to the manufacturer’s instructions (Gibco). Supernatants of 30 mL cultures with an initial density of 3 × 10^6^ cells/mL in Expi293 expression medium were collected 5 days after transfection, and recombinant antibodies were purified by affinity chromatography employing a Protein G column (Pierce, Thermo Fisher Scientific, Darmstadt, Germany) with an ÄKTA FPLC system (GE Healthcare Europe, Freiburg, Germany) [38]. The purity and identity of the isolated proteins was examined by SDS-PAGE, followed by Coomassie staining or immunoblot analysis with an HRP-conjugated anti-human IgG antibody (Sigma-Aldrich) and chemiluminescent detection. Protein concentrations were determined with a spectrophotometer (Nanodrop 1000; Thermo Fisher Scientific, Darmstadt, Germany), applying the calculated molecular mass and extinction coefficients. Typical yields ranged from 2.8 to 4.9 mg of purified NKAB proteins from 30 mL of initial culture supernatant. The binding of bispecific NKAB-EGFR and NKAB-ErbB2 molecules to NKG2D and their target antigens EGFR or ErbB2 on tumor cells was investigated by flow cytometry after incubating NKG2D- or NKAR-expressing immune cells and EGFR- or ErbB2-positive tumor cells with 12.5 nM of purified NKAB proteins, followed by APC-conjugated anti-human IgG secondary antibody (Jackson ImmunoResearch, Cambridgeshire, UK). A FACSCanto II flow cytometer (BD Biosciences) was used for analysis of the samples. Data were evaluated using FACSDiva version 6.1.3 and FlowJo version 10.8 software (BD Biosciences).

### 2.5. Generation of GL261 Glioblastoma Cells Expressing EGFR and ErbB2

Murine GL261 glioblastoma cells stably expressing human EGFR or ErbB2 were generated by transduction with VSV-G pseudotyped lentiviral vectors carrying the respective cDNAs. The resulting GL261/EGFR and GL261/ErbB2 cells were enriched via cell sorting with a FACSAria cell sorter using Alexa Fluor 488-conjugated anti-human EGFR (AY13) and Alexa Fluor 647-conjugated anti-human ErbB2 (24D2) antibodies (both Biolegend, Fell, Germany). GL261/ErbB2/EGFR cells co-expressing EGFR and ErbB2 were derived by the lentiviral transduction of GL261/ErbB2 cells with the EGFR-encoding vector.

### 2.6. Cytotoxicity Assays

Bispecific antibody-mediated redirection of NKAR-NK-92 and NKAR_RD-IL15-NK-92 cells to tumor cells expressing EGFR or ErbB2 was analyzed in flow cytometry-based cytotoxicity assays with target cells collected from regular adherent cell cultures (glioblastoma and breast carcinoma cells) or suspension cell cultures (K562 cells). Tumor cells were stained with Calcein Violet AM (CV) (CellTrace, Invitrogen, Thermo Fisher Scientific, Darmstadt, Germany) and co-cultured with effector cells at different effector to target cell (E/T) ratios for 3 h at 37 °C in the absence or presence of increasing concentrations of NKAB-EGFR or NKAB-ErbB2. After co-incubation, 100 µL of propidium iodide (PI) solution (1 µg/mL) were added to each sample, and cells were analyzed using an LSRFortessa cell analyzer (BD Biosciences). CV and PI double-positive cells were identified as dead target cells. To calculate specific cytotoxicity, spontaneous target cell lysis in the absence of effector cells was subtracted. Data were analyzed with FACSDiva software version 6.1.3. To assess cytotoxicity against mixed target cell cultures, GL261/EGFR cells stained with Far Red (CellTrace, Invitrogen) were mixed with CV-stained GL261/ErbB2 cells at a 1:1 ratio. Then, the mixed target cells were incubated for 3 h with effector cells at an E/T ratio of 5:1 in the absence or presence of 0.16 nM (25 ng/mL) of NKAB-EGFR or NKAB-ErbB2, or a combination of 0.08 nM (12.5 ng/mL) of each NKAB antibody. Dead cells within the different target cell populations were identified as Far Red and PI, or CV and PI double positive, respectively, and specific lysis was determined as described above.

### 2.7. Time-Lapse Microscopy

For real-time analysis of NKAB-mediated tumor cell-killing by primary lymphocytes, MDA-MB468 breast carcinoma cells, used as targets, were labeled with carboxyfluorescein succinimidyl ester (CFSE) (CellTrace, Invitrogen) and allowed to adhere to poly-L-lysine-coated glass bottom dishes (MatTek, Ashland, MA, USA) for 1 h. Then, PBMCs cultured for 3 days in medium containing IL-2 and IL-15, as described above, were stained with a BV421-conjugated antibody specific for CD56 and PE-conjugated anti-CD3 antibody (both BD Biosciences), and added to the CFSE-labeled target cells in the presence of 0.64 nM (100 ng/mL) of APC-conjugated NKAB-EGFR protein. Time-lapse imaging was conducted with a CQ1 Confocal Quantitative Image Cytometer (Yokogawa, Tokyo, Japan) at 40× magnification. Phase-contrast and fluorescent images were taken every 5 min by sample excitation with lasers at 405, 488, and 640 nm. Images were captured at four z-stacks and processed to maximum intensity projections (MIP).

### 2.8. Statistical Analysis

Quantitative data are represented as mean with standard deviation (SD). To determine statistical significance (*p* value < 0.05), a Welch’s unequal variances *t*-test was applied. All analyses were performed with Prism 9 (GraphPad Software, Boston, MA, USA).

## 3. Results

### 3.1. Generation of CAR-NK Cells Co-Expressing an NKG2D-Based CAR and an IL-15 Superagonist

**Figure 1:**
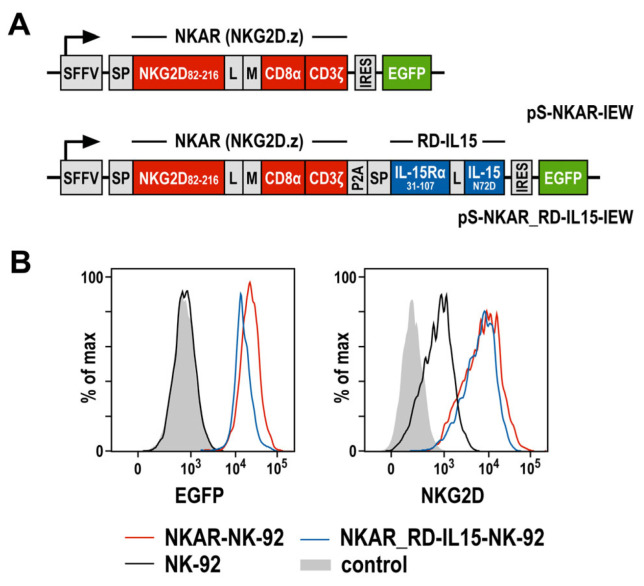
Generation of NK-92 cells expressing an NKG2D-based CAR and IL-15 superagonist RD-IL15. (**A**) Lentiviral transfer plasmids encoding the NKG2D-based CAR NKAR under the control of the spleen focus-forming virus promoter (SFFV). The NKAR encompasses an immunoglobulin heavy-chain signal peptide (SP), the extracellular domain of NKG2D (amino acid residues 82-216), a flexible (G_4_S)_2_ linker (L), a Myc-tag (M), a CD8α hinge region, and transmembrane and intracellular domains of CD3ζ. For co-expression of the IL-15 superagonist RD-IL15, a sequence consisting of a second signal peptide (SP), IL-15Rα sushi domain (amino acid residues 31-107), a peptide linker (L) and affinity-optimized IL-15_N72D_ was fused in frame to the NKAR sequence via a porcine teschovirus self-cleaving peptide (P2A). NKAR and RD-IL15 sequences are followed by an internal ribosome entry site (IRES) and enhanced green fluorescent protein (EGFP) cDNA. (**B**) The expression of EGFP (left), as well as NKG2D and the NKG2D-based CAR (right) by sorted NKAR-NK-92 and NKAR_RD-IL15-NK-92 cells was analyzed by flow cytometry, as indicated. Parental NK-92 cells were included for comparison. Unstained NK-92 cells served as a control.

To target NKG2D ligands present on the surface of tumor cells, we previously generated an NKG2D-based chimeric antigen receptor (termed NKAR), which is composed of an immunoglobulin heavy-chain signal peptide, the NKG2D extracellular domain followed by a flexible linker and a Myc-tag, connected to the transmembrane and intracellular domains of CD3ζ by a modified CD8α hinge region [27]. To further optimize the lentiviral CAR construct, a sequence encoding the IL-15 superagonist RD-IL15 was fused to the NKAR sequence via a porcine teschovirus self-cleaving peptide (P2A) (Figure 1A). RD-IL15 consists of a second immunoglobulin heavy-chain signal peptide, the sushi domain of IL-15Rα, and affinity-optimized IL-15_N72D_ [29,31]. VSV-G pseudotyped vector particles were generated and used for the transduction of NK-92 cells. The resulting NKAR-NK-92 and NKAR_RD-IL15-NK-92 cells were enriched by flow cytometric cell sorting based on their EGFP expression, and comparable levels of the NKAR on the cell surface were confirmed using an NKG2D-specific antibody (Figure 1B).

**Figure 2:**
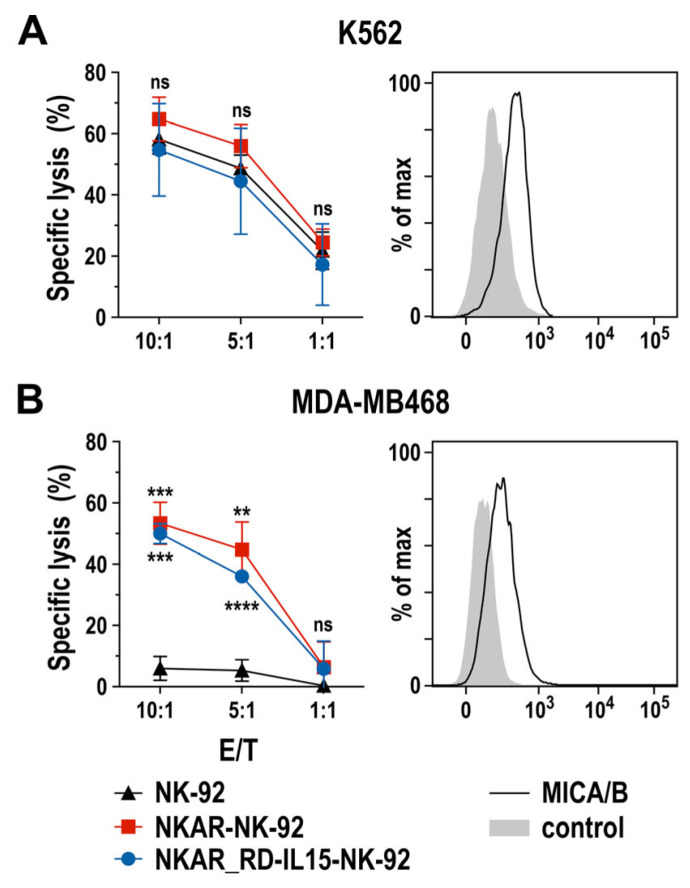
Cytotoxic activity of NKAR-engineered NK cells. Cell-killing of K562 erythroleukemia (**A**) and MDA-MB468 breast cancer cells (**B**) by NKAR-NK-92, NKAR_RD-IL15-NK-92 and parental NK-92 cells was investigated in flow cytometry-based cytotoxicity assays after co-incubation for 2 h at the indicated effector to target cell (E/T) ratios. Mean values ± SD are shown; n = 3 independent experiments. ****, *p* < 0.0001; ***, *p* < 0.001; **, *p* < 0.01; ns, *p* > 0.05 (not significant). The expression of NKG2D ligands on the surface of K562 and MDA-MB468 cells was confirmed by flow cytometry with MICA/B-specific antibody (black lines) as indicated. Unstained cells served as controls (filled gray areas).

To assess the specific cell-killing capacity of NKAR-expressing NK-92 cells against human tumor cells, we performed flow cytometry-based cytotoxicity assays using human K562 erythroleukemia cells and MDA-MB468 breast carcinoma cells as targets. Thereby, MHC class I-negative and intrinsically NK-sensitive K562 cells were lysed equally well by NKAR-NK-92, NKAR_RD-IL15-NK-92, and parental NK-92 cells after 2 h of co-incubation at different effector to target cell (E/T) ratios (Figure 2A), suggesting that, despite moderate NKG2D ligand expression by these targets, other activating receptors such as NKp30, NKp44, or NKp46 expressed by NK-92 cells contributed more to cell killing than NKG2D. Conversely, MDA-MB468 cells proved highly resistant to parental NK-92, but were readily lysed by NKAR-NK-92 and NKAR_RD-IL15-NK-92 cells (Figure 2B). Hence, this enhanced killing of the breast cancer cells can clearly be attributed to the recognition of NKG2D ligands such as MICA and MICB on the target cells’ surface by the NKG2D-based NKAR receptor [27].

These data demonstrate that the NKAR molecule is readily expressed in NK-92 cells, and mediates pronounced cytotoxicity against NKG2DL-positive tumor cells that are resistant to parental NK-92. Thereby, co-expression of the IL-15 superagonist did not negatively affect the activity of NKAR_RD-IL15-NK-92 when compared to NKAR-NK-92 cells.

### 3.2. Effect of RD-IL15 on NK Cell Activation and Proliferation

**Figure 3:**
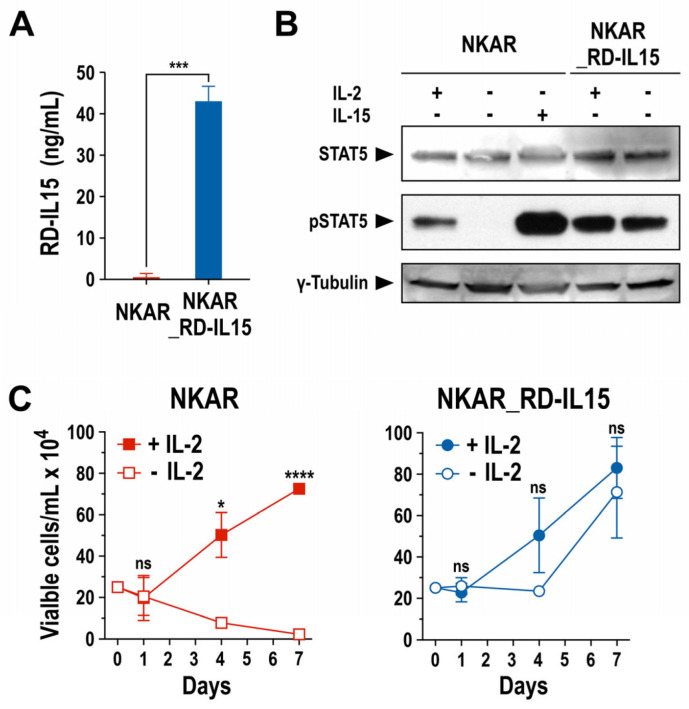
Biological activity of secreted IL-15 superagonist. (**A**) RD-IL15 secreted into the culture supernatant by NKAR_RD-IL15-NK-92 cells (blue bar) during a cultivation period of 3 days was measured by sandwich ELISA using an IL-15-specific capture antibody, and biotin-conjugated IL-15Rα-specific antibody and HRP-coupled streptavidin for detection. Recombinant His-tagged RD-IL15 was used as a standard for quantification. Culture supernatant from NKAR-NK-92 cells (red bar) served as a control. Mean values ± SD are shown; n = 3. ***, *p* < 0.001. (**B**) Lysates of NKAR-NK-92 and NKAR_RD-IL15-NK-92 cells cultured for 24 h in medium with or without IL-2 (100 IU/mL) or IL-15 (20 ng/mL) were subjected to SDS-PAGE and immunoblotting with antibodies specific for total and phosphorylated STAT5. γ-Tubulin served as a loading control. Uncropped images of the blots are shown in Supplementary Figure S7. (**C**) Proliferation of NKAR-NK-92 (left) and NKAR_RD-IL15-NK-92 cells (right) grown in medium with (filled symbols) or without (open symbols) IL-2 (100 IU/mL) for 7 days. Cell numbers were determined on days 0, 1, 4, and 7. Mean values ± SD are shown; n = 3 independent experiments. ****, *p* < 0.0001; *, *p* < 0.05; ns, *p* > 0.05 (not significant).

Next, we analyzed production of the RD-IL15 protein by NKAR_RD-IL15-NK-92 cells and functionality of the cytokine. A sandwich ELISA experiment confirmed that the engineered NK cells indeed produced the IL-15 superagonist constitutively, and secreted it at high levels into the culture supernatant (Figure 3A). Subsequently, potential autocrine stimulation of NKAR_RD-IL15-NK-92 cells by RD-IL15 was investigated by analyzing the phosphorylation of STAT5 as a downstream mediator of IL-2R/IL-15R signaling. The NK cells were grown overnight in the presence or absence of IL-2 or IL-15, before preparation of whole cell lysates for immunoblot analysis of phosphorylated STAT5 (pSTAT5). As expected, pSTAT5 was detected in NKAR-NK-92 cells after culture with exogenous IL-2 or IL-15, while no STAT5 activation was observed in the absence of the cytokines. In contrast, irrespective of the addition of exogenous cytokines, pSTAT5 levels remained high in NKAR_RD-IL15-NK-92 cells (Figure 3B), indicating autocrine activation of the cells by the ectopically expressed IL-15 superagonist.

This was confirmed in proliferation assays that demonstrated continuous expansion of NKAR_RD-IL15-NK-92 cells after removal of IL-2 from the growth medium, while the numbers of NKAR-NK-92 cells lacking the IL-15 superagonist quickly decreased in the absence of exogenous IL-2 (Figure 3C). Likewise, cell viability was strictly dependent on the addition of IL-2 in the case of NKAR-NK-92 cells, but not for NKAR_RD-IL15-NK-92 (Supplementary Figure S1A). In surface marker analysis, NKAR_RD-IL15-NK-92 cells displayed lower levels of activating NKp46 in comparison to NKAR-NK-92 cells, but they also showed an increased expression of activating NKp44 and a marked reduction in inhibitory NKG2A (Supplementary Figure S1B). Nevertheless, there was no difference in the expression of the early activation marker CD69 and no upregulation of TIGIT as a marker for exhaustion. In addition to its autocrine effects on NKAR_RD-IL15-NK-92 cells, secreted RD-IL15 also supported, in a paracrine fashion, the growth of NKAR-NK-92 cells lacking the IL-15 superagonist in transwell assays (Supplementary Figure S2A), and enhanced the activity of neighboring primary immune cells, indicated by the enhanced cytotoxicity of innate bystander lymphocytes and increased proliferation of cytotoxic T cells (Supplementary Figure S2B,C).

### 3.3. Generation of a Bispecific Killer Cell Engager Binding to NKG2D and Epidermal Growth Factor Receptor

**Figure 4:**
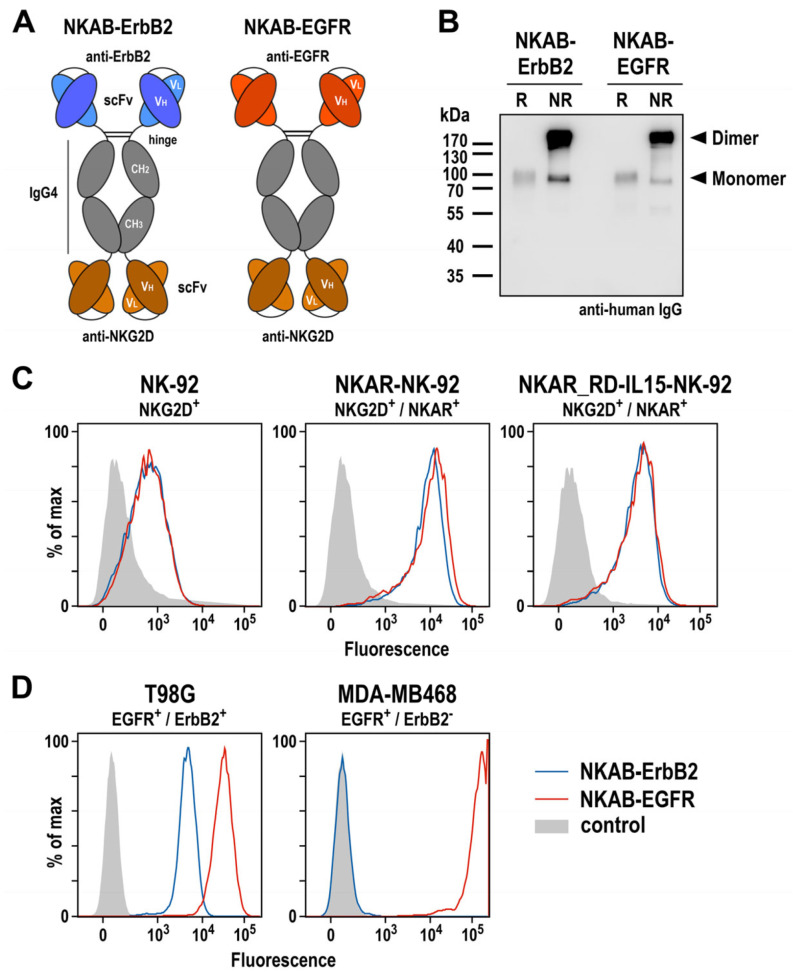
Expression and functional characterization of bispecific antibodies. (**A**) Schematic representation of NKAB-ErbB2 and NKAB-EGFR molecules that consist of an N-terminal scFv antibody fragment binding to ErbB2 or EGFR as indicated, followed by hinge, CH2 and CH3 domains of human IgG4, a (G_4_S)_2_ linker, and an NKG2D-specific C-terminal scFv antibody fragment. Disulfide bridges facilitating the formation of homodimers are indicated by lines. (**B**) Immunoblot analysis of purified NKAB-ErbB2 and NKAB-EGFR antibodies after SDS-PAGE under reducing (R) and non-reducing (NR) conditions. The recombinant proteins were detected using an HRP-conjugated anti-human IgG antibody. NKAB monomers and dimers are indicated by arrowheads. An uncropped image of the blot is shown in Supplementary Figure S8. The binding of purified NKAB-EGFR (red lines) and NKAB-ErbB2 (blue lines) to parental and NKAR-expressing NK-92 cells (**C**), as well as EGFR and ErbB2 double-positive T98G glioblastoma and EGFR-expressing MDA-MB468 breast carcinoma cells (**D**), was investigated by flow cytometry. Cells only incubated with secondary antibody served as controls (gray areas).

To specifically redirect lymphocytes endogenously expressing NKG2D or engineered with an NKG2D-based CAR to tumor cells with low or absent NKG2D ligand expression, we previously described different designs for bispecific molecules, which all reflect the structure and approximate size of IgG antibodies, but can simultaneously interact with NKG2D and the tumor-associated antigen ErbB2 (HER2) via respective single-chain fragment variable (scFv) antibodies connected by hinge, CH2, and CH3 domains of IgG4 [27]. Following the design of such an NKAB-ErbB2 molecule carrying the tumor-targeting domain at the N-terminus and the NKG2D-specific domain at the C-terminus, here, we generated a similar NKAB-EGFR antibody for the redirection of effector lymphocytes to tumor cells overexpressing epidermal growth factor receptor (EGFR) (Figure 4A). For specific tumor targeting, this molecule contains an scFv antibody fragment derived from monoclonal antibody 225, which has the same antigen binding site as the chimeric antibody cetuximab [37]. NKAB-EGFR and NKAB-ErbB2 were expressed as secreted proteins at high yields in transiently transfected Expi293F cells, and purified from culture supernatants by Protein G affinity chromatography. SDS-PAGE (Supplementary Figure S3) and immunoblot analysis of the purified antibodies with an IgG-specific antibody (Figure 4B) confirmed the identity of the recombinant proteins and their production predominantly as disulfide-linked homodimers, detected under non-reducing conditions.

Specific binding of the NKAB molecules to NKG2D-expressing effector cells and antigen-positive tumor cells was investigated by flow cytometry. Thereby, a strong and comparable binding of NKAB-EGFR and NKAB-ErbB2 to both, NKAR-NK-92 and NKAR_RD-IL15-NK-92 cells carrying the NKG2D-based CAR was detected, while the signals obtained with parental NK-92 cells were less pronounced due to the only moderate endogenous NKG2D expression of these cells (Figure 4C). The tumor-targeting domains of the bispecific antibodies were also functional, indicated by a strong binding of NKAB-EGFR and less pronounced binding of NKAB-ErbB2 to T98G glioblastoma cells, which express high levels of EGFR and more moderate levels of ErbB2. In contrast, only NKAB-EGFR displayed binding to MDA-MB468 breast carcinoma cells, which highly overexpress EGFR but are negative for ErbB2.

Collectively, these results show that, like the previously described NKAB-ErbB2 antibody, the newly generated NKAB-EGFR molecule is produced as a homodimeric, tetravalent protein which specifically binds to NKG2D and its cognate tumor-associated antigen.

### 3.4. Tumor-Specific Redirection of NKAR-Expressing NK-92 and Peripheral Blood-Derived NK Cells by NKAB-EGFR Antibody

**Figure 5:**
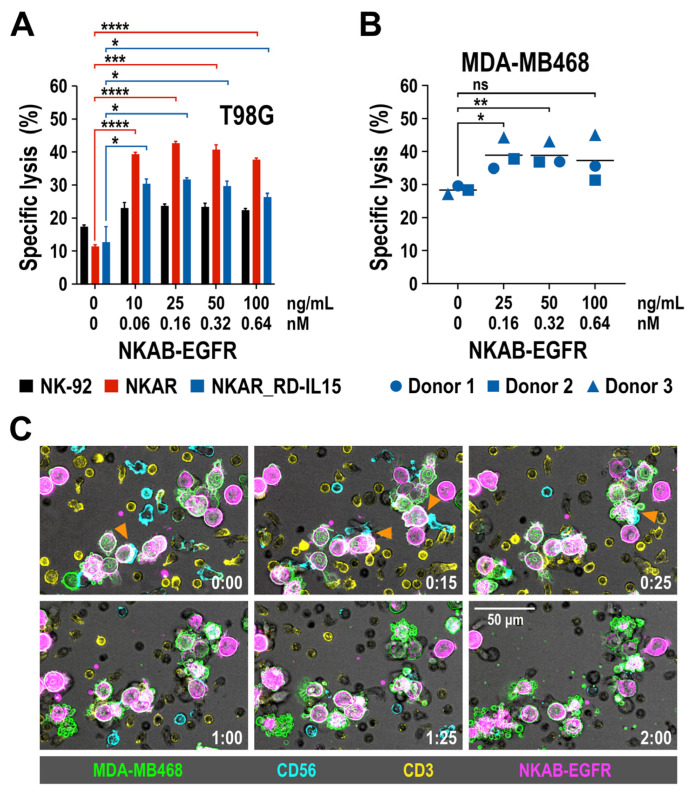
Tumor-specific redirection of NKAR-NK-92, NKAR_RD-IL15-NK-92 and peripheral blood-derived primary NK cells by NKAB-EGFR. (**A**) The effect of NKAB-EGFR on the specific cytotoxicity of NKAR-NK-92 (red bars) and NKAR_RD-IL15-NK-92 cells (blue bars) against T98G glioblastoma cells was determined in flow cytometry-based cytotoxicity assays after co-incubation at an effector to target cell (E/T) ratio of 5:1 for 3 h in the absence or presence of increasing NKAB-EGFR concentrations. Parental NK-92 cells were included for comparison (black bars). Mean values ± SD are shown; n = 3 independent experiments. ****, *p* < 0.0001; ***, *p* < 0.001; *, *p* < 0.05. (**B**) Cytotoxicity of donor-derived primary NK cells against MDA-MB468 breast carcinoma cells in the absence or presence of increasing NKAB-EGFR concentrations after 3 h of co-incubation at an E/T ratio of 20:1. Individual data points for NK cells from three independent donors are shown. Mean values are indicated by lines. **, *p* < 0.01; *, *p* < 0.05; ns, *p* > 0.05 (not significant). (**C**) Interaction of primary lymphocytes with breast cancer cells in the presence of the bispecific NKAB-EGFR antibody. Peripheral blood mononuclear cells from a healthy donor were labeled with antibodies specific for CD56 (cyan) and CD3 (yellow), followed by co-incubation with CFSE-labeled MDA-MB468 cells (green) in the presence of 0.64 nM (100 ng/mL) of APC-conjugated NKAB-EGFR (magenta). Phase-contrast and fluorescent images were taken every 5 min at 40× magnification with a CQ1 Confocal Quantitative Image Cytometer. Serial images of a representative field taken at the indicated time points (hours:minutes) are shown. Orange arrowheads point to initial contacts between CD56-positive NK cells and NKAB-EGFR-opsonized tumor cells. Subsequent target cell death is indicated by membrane blebbing and the formation of apoptotic bodies. Scale bar: 50 μm.

We previously demonstrated that recombinant NKAB-ErbB2 mediated high and tumor-cell-specific cytotoxicity of effector cells endogenously expressing NKG2D or engineered with the NKAR receptor [27]. To test whether this is also true for the newly generated NKAB-EGFR molecule, EGFR-positive T98G glioblastoma cells were co-cultured with NKAR-NK-92 and NKAR_RD-IL15-NK-92 cells for 3 h at an E/T ratio of 5:1 in the absence or presence of increasing concentrations of purified NKAB-EGFR antibody, before the specific lysis of target cells was determined by flow cytometry. Parental NK-92 cells were included for comparison. Thereby, NKAB-EGFR significantly increased target cell killing by NKAR-NK-92 and NKAR_RD-IL15-NK-92 cells in a dose-dependent manner, from 11.3% and 12.7% in the absence of the bispecific antibody up to a maximum of 42.7% and 31.7% lysis, respectively, which was reached at an NKAB-EGFR concentration of 0.16 nM (25 ng/mL) (Figure 5A). The cytotoxic activity of NKAR-NK-92 and NKAR_RD-IL15-NK-92 cells slightly decreased again if more NKAB-EGFR was present, likely due to a competition of productive cross-linking of effector and target cells by free antibody molecules. The addition of NKAB-EGFR also enhanced cell killing by parental NK-92 cells, albeit to a much lower degree, which can be explained by the cells’ moderate expression of endogenous NKG2D (see Figure 1B).

Next, we analyzed the effect of NKAB-EGFR on the lytic activity of primary NK cells. pNK cells were isolated from peripheral blood mononuclear cells (PBMC) of three healthy donors and expanded for 3 days in medium containing IL-2 and IL-15, before analysis by flow cytometry. A >95% pure population of CD56^+^ CD3^-^ NK cells was obtained from each donor, with all of the cells positive for NKG2D, and the majority of them also expressing CD16 (Supplementary Figure S4). pNK cells were then co-cultured for 3 h at an E/T ratio of 20:1 with EGFR-expressing MDA-MB468 breast cancer cells in the absence or presence of increasing amounts of NKAB-EGFR. Subsequent determination of specific target cell lysis revealed that, in the case of primary NK cells, the addition of low NKAB-EGFR concentrations of 0.16 or 0.32 nM (25 or 50 ng/mL) already significantly enhanced cytotoxicity against the tumor cells (Figure 5B). To investigate the kinetics of NKAB-EGFR-mediated tumor cell killing by primary lymphocytes, a time-lapse microscopy experiment was performed. PBMCs from a healthy donor were cultured to preferentially stimulate T and NK cells, and then incubated with MDA-MB468 breast carcinoma cells in the presence of APC-conjugated NKAB-EGFR. Thereby, the tumor cells were quickly opsonized by the bispecific antibody, enabling interaction predominantly with CD56-positive NK cells, which resulted in target cell lysis, as indicated by membrane blebbing and the formation of apoptotic bodies, within approximately one hour after initial contact (Figure 5C).

Taken together, these data demonstrate the functionality of NKAB-EGFR as a bispecific killer cell engager, mediating effective tumor cell killing by NKAR-engineered effector cells, as well as primary NK cells, which endogenously express NKG2D.

### 3.5. Activity of NKAB-EGFR and NKAB-ErbB2 against Tumor Cells with Heterogeneous Target Antigen Expression

**Figure 6:**
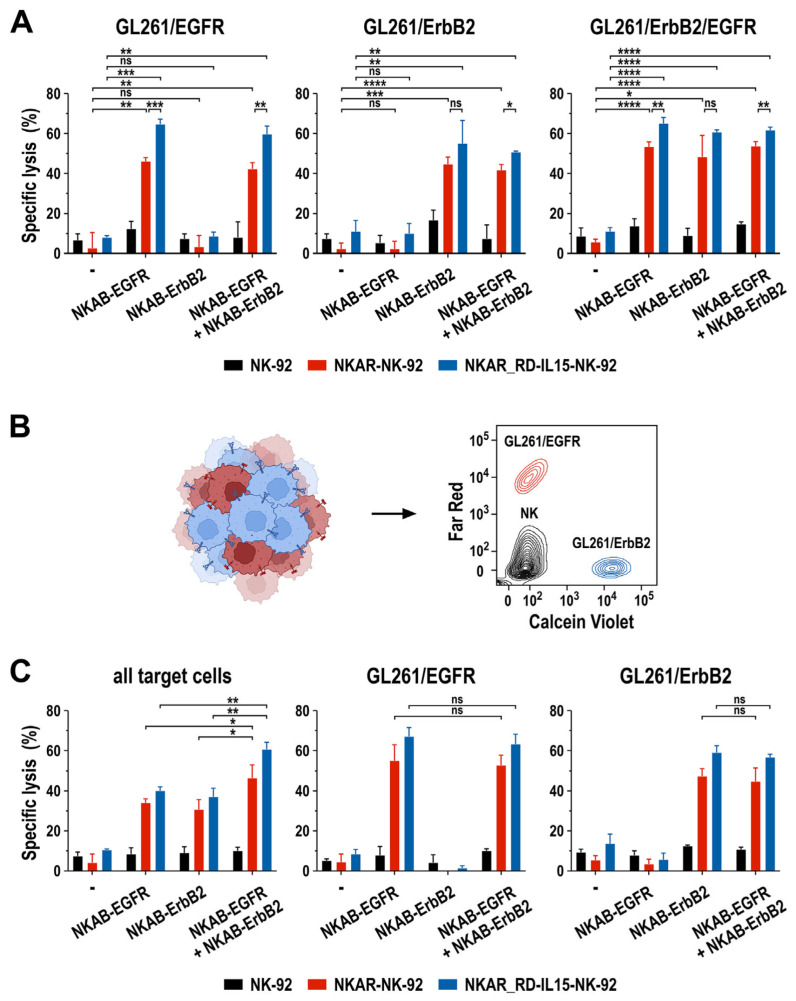
Activity of NKAB-EGFR and NKAB-ErbB2 against glioblastoma cells with heterogeneous target antigen expression. (**A**) Specific cytotoxicity of NKAR-NK-92 (red bars) and NKAR_RD-IL15-NK-92 cells (blue bars) against murine GL261 glioblastoma cells homogeneously expressing human EGFR, ErbB2, or both target antigens in the absence or presence of 0.16 nM (25 ng/mL) of NKAB-EGFR or NKAB-ErbB2, or a combination of 0.08 nM (12.5 ng/mL) of each molecule. Effector and target cells were co-cultured at an E/T ratio of 5:1 for 3 h. Parental NK-92 cells were included for comparison (black bars). (**B**) To assess the activity of the NKAB molecules against tumor cells with heterogeneous antigen expression, GL261/EGFR cells stained with Far Red (red) and GL261/ErbB2 cells stained with Calcein Violet (blue) were mixed at a 1:1 ratio before exposure to NK cells and bispecific antibodies. A representative contour plot identifying the different cell types by flow cytometry is shown on the right. (**C**) Specific cytotoxicity of NKAR-NK-92 (red bars) and NKAR_RD-IL15-NK-92 cells (blue bars) against mixed GL261/EGFR and GL261/ErbB2 glioblastoma cell cultures in the absence or presence of NKAB-EGFR or NKAB-ErbB2, or a combination of both molecules. The cell-killing experiments were performed as described in (**A**). Parental NK-92 cells were included for comparison (black bars). The panels depict the extent of specific lysis of all glioblastoma cells in the assay (left), or the individual GL261/EGFR (middle) and GL261/ErbB2 subpopulations (right). Mean values ± SD are shown in (**A**,**C**); n = 3 independent experiments. ****, *p* < 0.0001; ***, *p* < 0.001; **, *p* < 0.01; *, *p* < 0.05; ns, *p* > 0.05 (not significant).

In particular for solid tumors such as glioblastoma, antigen-specific immunotherapy can be complicated by heterogeneous target antigen expression and the treatment-induced selection of antigen-loss variants [37,39]. To investigate the activity of NKAR-expressing NK-92 cells in combination with bispecific NKAB molecules in such a setting, we established an in vitro model based on murine GL261 glioblastoma cells lentivirally transduced to express one or both of the human EGFR and ErbB2 target antigens on their surface, allowing for the specific binding of the bispecific killer cell engagers (Supplementary Figure S5). First, the cytotoxic activity of NKAR-NK-92 and NKAR_RD-IL15-NK-92 cells against homogeneous cultures of GL261/EGFR, GL261/ErbB2, and GL261/ErbB2/EGFR glioblastoma cells in the presence of NKAB-EGFR or NKAB-ErbB2, or a combination of both molecules, was tested. For these assays, we used concentrations of 0.16 nM (25 ng/mL) of the individual NKAB proteins, or a combination of 0.08 nM (12.5 ng/mL) of each antibody, identified beforehand as optimal concentrations in titration experiments (Supplementary Figure S6). On their own, only a modest baseline cytotoxicity of NKAR-expressing NK-92 cells against the different glioblastoma cell lines was observed, which was similar to that of parental NK-92 cells. In contrast, the addition of NKAB-EGFR resulted in the high and specific lysis of GL261 cells expressing EGFR, while NKAB-ErbB2 mediated specific cell killing against ErbB2-expressing targets (Figure 6A, left and middle). GL261/ErbB2/EGFR cells expressing both antigens were eliminated equally well in the presence of either NKAB molecule alone, or a combination of NKAB-EGFR and NKAB-ErbB2 (Figure 6A, right).

To mimic a heterogeneous tumor cell population, in the next set of experiments, GL261/EGFR and GL261/ErbB2 cells were mixed at a 1:1 ratio, and then co-cultured for 3 h with NKAR-NK cells in the absence or presence of 0.16 nM (25 ng/mL) of the individual NKAB proteins, or a combination of 0.08 nM (12.5 ng/mL) of NKAB-EGFR and NKAB-ErbB2. To distinguish the different cell types in the assay, GL261/EGFR were labeled with Far Red and GL261/ErbB2 cells with Calcein Violet before initiating the co-culture, while NK cells remained unstained (Figure 6B). In contrast to the comparable activity of NKAB-EGFR, NKAB-ErbB2 or a combination of the NKAB molecules seen with GL261 cells expressing both target antigens (Figure 6A, right), treatment of the heterogeneous tumor cell culture of GL261/EGFR and GL261/ErbB2 cells with NKAR-NK cells and a single NKAB antibody resulted in much less pronounced target cell killing than the combination of NKAB-EGFR and NKAB-ErbB2 (Figure 6C, left). This was due to NKAB-EGFR and NKAB-ErbB2 only mediating the selective killing of the tumor cell subpopulation within the mix that expressed the cognate tumor antigen, as evidenced by analysis of the individual target cell subtypes (Figure 6C, middle and right).

These data demonstrate that the antitumor effects mediated by NKAB-EGFR and NKAB-ErbB2 through interaction with NKAR-NK cells are highly specific. Nevertheless, when applied in combination, tumor cell populations with heterogeneous antigen expression are also effectively eliminated, which is important to prevent immune escape.

## 4. Discussion

Similar to bispecific antibodies like blinatumomab, that selectively redirect T lymphocytes to cancer cells via binding to CD3 [40], bispecific and trispecific molecules, which recruit innate killer cells to the tumor by engaging CD16, natural cytotoxicity receptors, or NKG2D, are gaining increasing interest and clinical relevance [20,41,42,43]. Using cellular in vitro models of glioblastoma and other solid tumors, we evaluated in our study the activity of a novel bispecific antibody termed NKAB-EGFR, which employs the binding domain of the EGFR-specific antibody cetuximab for tumor targeting and an NKG2D-interacting antibody fragment for NK-cell recruitment [36,37]. The two binding domains were linked via an IgG4 Fc region that enabled its expression as a homodimeric protein with a structure similar to that of natural IgG. Like the previously described NKAB-ErbB2 protein, which is selective for the receptor tyrosine kinase ErbB2 (HER2) [27], NKAB-EGFR enhanced the activity of peripheral blood NK cells from healthy donors against tumor cells expressing the cognate target antigen. More pronounced antitumor activity was observed when NKAB-EGFR and NKAB-ErbB2 were combined with NK-92 cells carrying an NKG2D-based CAR. While these NKAR-NK-92 and NKAR_RD-IL15-NK-92 cells were already active on their own against tumor cells expressing NKG2D ligands, specific lysis of murine glioblastoma cells employed as a model for NKG2DL-negative targets was only observed in the presence of a suitable NKAB molecule. The latter finding will likely be important in a clinical setting, where cancer cells can evade NKG2D-mediated immune surveillance by the proteolytic shedding or downregulation of NKG2DL [12,13,14,15].

Recently, safety concerns emerged regarding T cells engineered with an NKG2D-CAR similar to the NKAR receptor used in our study. While still under active clinical development for different hematological malignancies [11], fatal side effects occurred in the KEYNOTE-B79 phase Ib trial with allogeneic NKG2D-CAR T cells in colorectal cancer (NCT04991948; clinicaltrials.gov), and patient recruitment was discontinued. In contrast to T cells, NK and CAR-NK cells can be applied in an allogeneic setting without inducing graft-versus-host disease [44], and initial clinical data on CAR-NK cells indicate a markedly reduced risk of inducing cytokine release syndrome (CRS) or immune effector cell-associated neurotoxicity syndrome (ICANS) [45]. The results from a pilot study in colorectal cancer patients suggest that this safety advantage also extends to NKG2D-CAR engineered NK cells [46]. Here, we used NKG2D-CAR effector cells derived from the continuously expanding human NK cell line NK-92. Unmodified and CAR-engineered NK-92 cells were safely applied in early-phase clinical trials, with signs of clinical activity observed in some of the treated patients [47,48,49]. While, in a clinical setting, these cells are irradiated before application, the ensuing lack of in vivo expansion and long-term engraftment may be compensated by repeated treatments [50], and can be considered an additional safety feature, especially when targeting tumor-associated antigens like EGFR that are also expressed in healthy tissues.

Earlier work showed that the dependence of NK-92 on exogenous IL-2 for growth and functional activity can be addressed by the genetic modification of the cells with constructs encoding IL-2 or IL-15 [51,52]. Following a similar strategy, here, we co-expressed the NKAR receptor together with the IL-15 superagonist RD-IL15, which resulted in the autocrine activation of the NK cells and a preservation of their proliferative potential in the absence of IL-2. While it has been reported that prolonged stimulation with IL-15 may result in the exhaustion of NK cells [53], a surface marker analysis of NKAR_RD-IL15-NK-92 cells did not indicate an exhausted phenotype when compared to NKAR-NK-92 cells lacking the IL-15 superagonist (see Supplementary Figure S1B). RD-IL15 was secreted by NKAR_RD-IL15-NK-92 cells in amounts sufficient to also support co-cultured NKAR-NK-92 cells that did not produce RD-IL15 themselves, and to activate co-cultured primary lymphocytes in transwell assays (see Supplementary Figure S2). The latter effect could be relevant in an in vivo setting, complementing the NKG2D-CAR- and NKAB-mediated cytotoxicity of IL-15-producing NKAR-NK-92 cells by also enhancing the antitumor activity of endogenous immune cells in the tumor microenvironment. Furthermore, IL-15 can upregulate NKG2D expression [54], which may increase the additional recruitment of endogenous NK and CD8^+^ T cells by bispecific NKAB antibodies. An analysis of tumor organoid models complemented with primary immune cells and in vivo experiments in immunocompetent mouse tumor models may be helpful to further investigate the modulation of the tumor immune microenvironment by NKAR_RD-IL15-NK-92 cells and potential exhaustion of the CAR-NK cells in a setting that more closely reflects the clinical situation [55].

NKG2D-CAR-engineered NKAR-NK-92 and NKAR_RD-IL15-NK-92 cells both displayed effective NKAB-EGFR- and NKAB-ErbB2-mediated cytotoxicity against glioblastoma cells. This was also the case when treating mixed cultures of EGFR- and ErbB2-expressing cancer cells that served as a model for heterogeneous target antigen expression, which is often observed in solid tumors and can contribute to their escape from immunotherapy [56,57]. Co-cultures of GL261/EGFR and GL261/ErbB2 cells were only effectively lysed if both NKAB molecules were present, whereas NKAB-EGFR and NKAB-ErbB2 alone merely mediated the killing of the respective target cell subpopulation. This suggests a combination of the same NKAR-expressing effector cells with NKAB molecules of different specificities as an approach for personalized cancer therapy, which can also take into account the treatment-induced selection of cancer cell subpopulations that lost the initial target antigen.

## 5. Conclusions

As a proof of concept, in this in vitro study, we investigated the combined activity of NKG2D-CAR engineered NK cells and bispecific killer cell engagers targeting EGFR and ErbB2. On their own, the NKAB molecules enhanced the antitumor activity of primary NK cells that did not harbor an NKG2D-CAR but endogenously expressed NKG2D. Also, NKG2D-CAR-engineered NKAR-NK-92 and NKAR_RD-IL15-NK-92 cells effectively lysed tumor cells that expressed natural NKG2D ligands in the absence of the bispecific killer cell engagers. However, NKG2DL-negative tumor cells were only lysed when NKAB-EGFR and NKAB-ErbB2 antibodies were applied together with the NKG2D-CAR NK cells. This demonstrates the added benefit of the combination therapy. Nevertheless, it will be important to further validate our findings in more sophisticated model systems such as primary glioblastoma cells, patient-derived organoids, and mouse tumor models, which are now planned as the next step.

## Data Availability

Data supporting the findings of this study are available within the article, the Supplementary Materials, or from the corresponding author upon reasonable request.

## Supplementary Materials

The following supporting information can be downloaded at: https://www.mdpi.com/article/10.3390/cells13030246/s1, Figure S1: Viability and surface marker expression of NKAR-NK-92 and NKAR_RD-IL15-NK-92 cells; Figure S2: RD-IL15-mediated activation of bystander immune cells; Figure S3: Purification of recombinant NKAB molecules; Figure S4: Purity of ex vivo expanded peripheral blood NK cells; Figure S5: Binding of NKAB-EGFR and NKAB-ErbB2 antibodies to GL261 glioblastoma cells expressing EGFR, ErbB2 or both target antigens; Figure S6: Lysis of GL261/EGFR and GL261/ErbB2 cells by NKAR-NK-92 and NKAR_RD-IL15-NK-92 cells in the presence of increasing concentrations of NKAB-EGFR or NKAB-ErbB2. Figure S7: Uncropped images of the immunoblots shown in Figure 3B. Figure S8: Uncropped image of the immunoblot shown in Figure 4B.
