# Supplementary material for: Dual Targeting of Glioblastoma Cells with Bispecific Killer Cell Engagers Directed to EGFR and ErbB2 (HER2) Facilitates Effective Elimination by NKG2D-CAR-Engineered NK Cells
Source: Cells. 2024 Jan 28;13(3):246. doi: 10.3390/cells13030246 (PMC10854564; doi:10.3390/cells13030246)
Supplement: Supplementary file 1 [file cells-13-00246-s001.zip › cells-2773275-supplementary.pdf]

Supplementary Materials

Dual Targeting of Glioblastoma Cells with Bispecific Killer Cell Engagers Directed to EGFR and ErbB2 (HER2) Facilitates Effective Elimination by NKG2D-CAR-Engineered NK Cells

Anne Kiefer, Maren Prüfer, Jasmin Röder, Jordi Pfeifer Serrahima,

Malena Bodden, Ines Kühnel, Pranav Oberoi and Winfried S. Wels

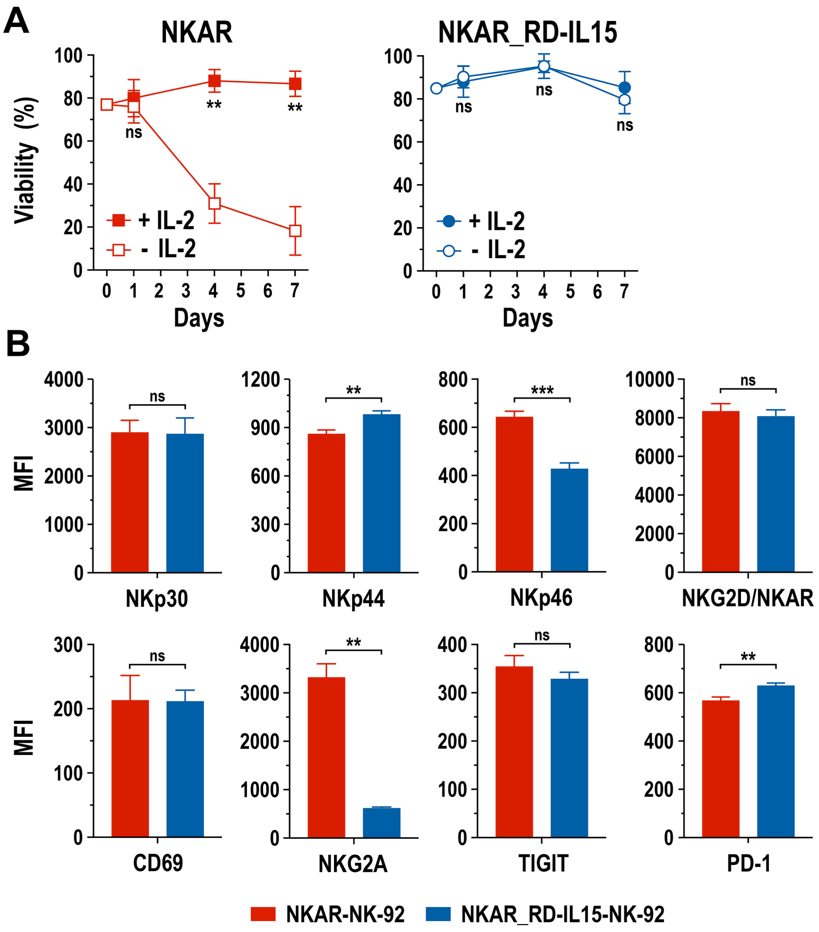

Figure S1. (A) Viability of NKAR-NK-92 (left) and NKAR_RD-IL15-NK-92 cells (right) grown in medium with (filled symbols) or without (open symbols) IL-2 for 7 days. The proportion of viable cells was determined on days 0, 1, 4 and 7. Mean values ± SD are shown; n=3 independent experiments. **, $p < 0.01$; ns, $p > 0.05$ (not significant). **(B)** Surface marker analysis of NKAR-NK-92 and NKAR_RD-IL15-NK-92 cells. Cell surface expression of the indicated activating and inhibitory receptors by NKAR-NK-92 and NKAR_RD-IL15-NK-92 cells was analyzed by flow cytometry. MFI: mean fluorescence intensity (geometric mean). Mean values ± SD are shown; n=3; ***, $p < 0.001$; **, $p < 0.01$; ns, $p > 0.05$ (not significant).

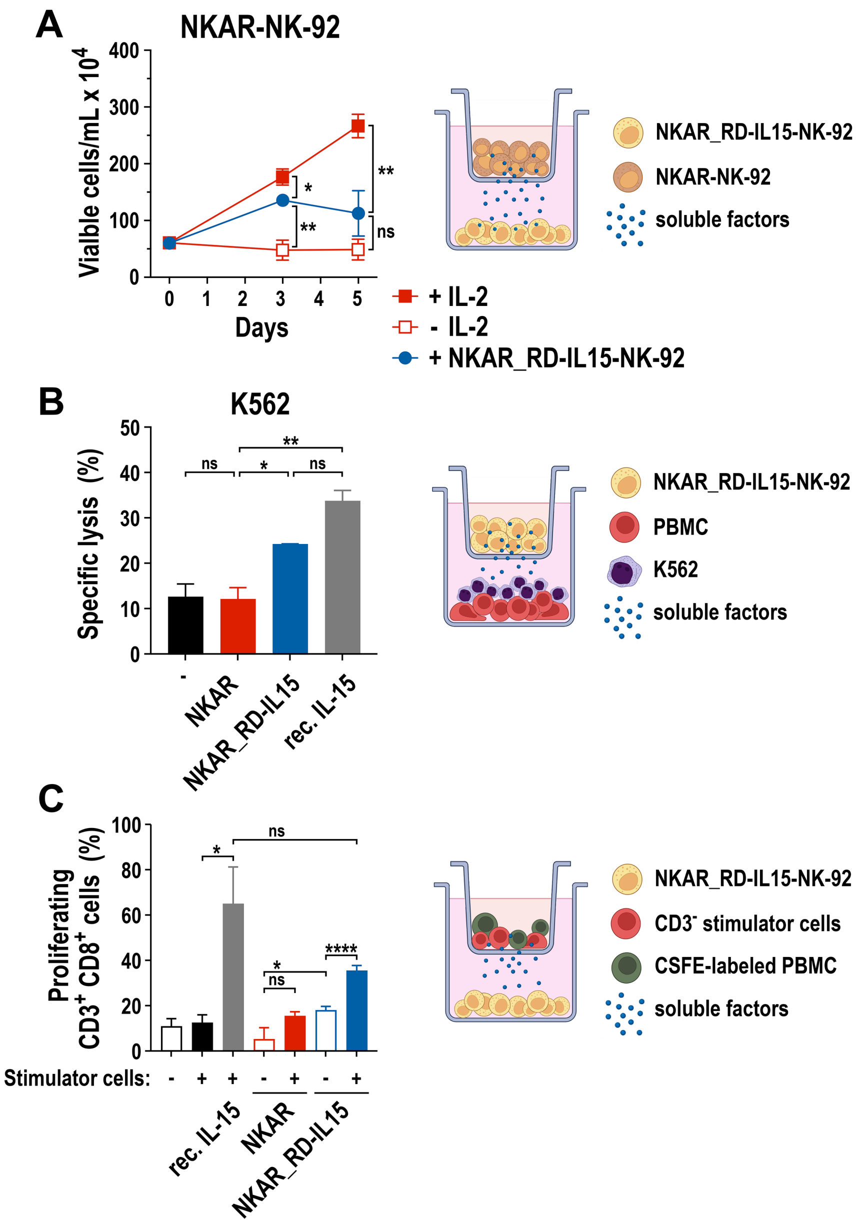

Figure S2. (A) Effect of soluble RD-IL15 on NKAR-NK-92 cells. Proliferation of NKAR-NK-92 grown for 5 days on their own in transwell inserts in medium with or without 100 IU/mL IL-2 (filled and open red squares), or grown in medium without IL-2 but exposed to soluble factors from co-cultured NKAR_RD-IL15-NK-92 cells that produce the IL-15 superagonist (filled blue circles). Mean values ± SD are shown; n=3. **, $p < 0.01$; *, $p < 0.05$; ns, $p > 0.05$ (not significant). **(B)** Effect of RD-IL15 on the cytotoxic activity of innate bystander lymphocytes. Peripheral blood mononuclear cells (PBMC) from healthy donors were grown for 16 hours in medium lacking IL-2, while exposed to soluble factors from NKAR_RD-IL15-NK-92 cells added in a transwell insert. Then, K562 cells were mixed with the stimulated PBMCs at an E/T ratio of 20:1 as schematically shown on the right. After 2 hours of co-incubation, specific lysis of K562 cells by the PBMCs was determined in a flow cytometry-based cytotoxicity assay (left). **(C)** Stimulation of cytotoxic T cells by RD-IL15. In a mixed lymphocyte reaction, CFSE-labeled PBMCs were seeded in transwell inserts and cultured with or without irradiated $CD3^-$ stimulator cells of an unrelated second donor, while exposed to secreted factors from NKAR_RD-IL15-NK-92 cells kept in the bottom well as schematically shown on the right. After 7 days of co-culture, the proportion of proliferating $CD8^+$ T lymphocytes was determined by flow cytometry according to their decreasing CFSE signal (left). In (B) and (C), control samples were kept without engineered NK cells, or exposed to 20 ng/mL of recombinant IL-15 or NKAR-NK-92 cells lacking RD-IL15 as indicated. Mean values ± SD are shown; n=3 independent donors. ****, $p < 0.0001$; **, $p < 0.01$; *, $p < 0.05$; ns, $p > 0.05$ (not significant).

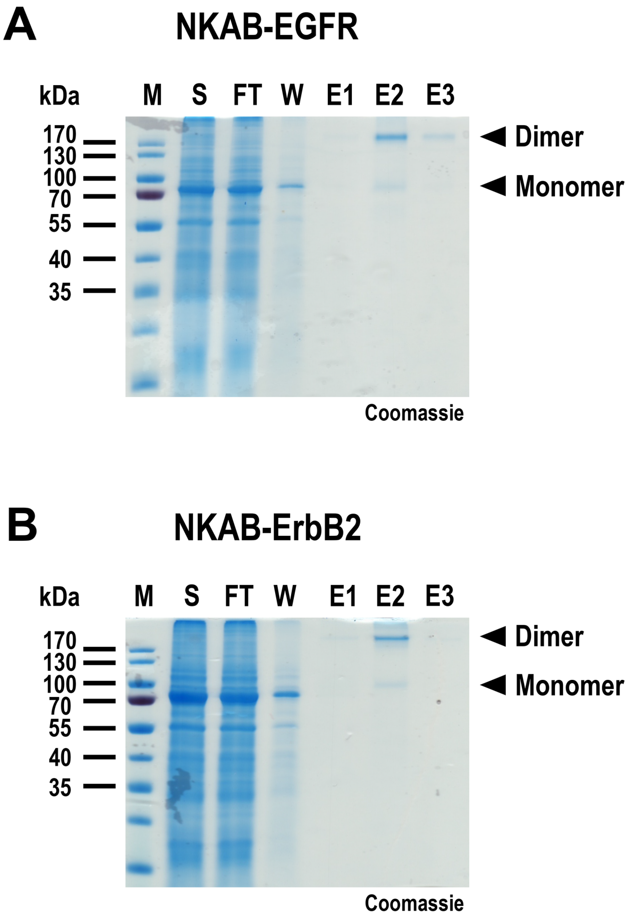

Figure S3. Purification of recombinant NKAB molecules. SDS-PAGE analysis of **(A)** NKAB-EGFR and **(B)** NKAB-ErbB2 purified from culture supernatants of transiently transfected Expi293F cells by Protein G affinity chromatography. Proteins were separated under non-reducing conditions. NKAB monomers and dimers are indicated by arrowheads. M, prestained marker; S, culture supernatant; FT, flow through; W, wash fraction; E1, E2, E3, elution fractions.

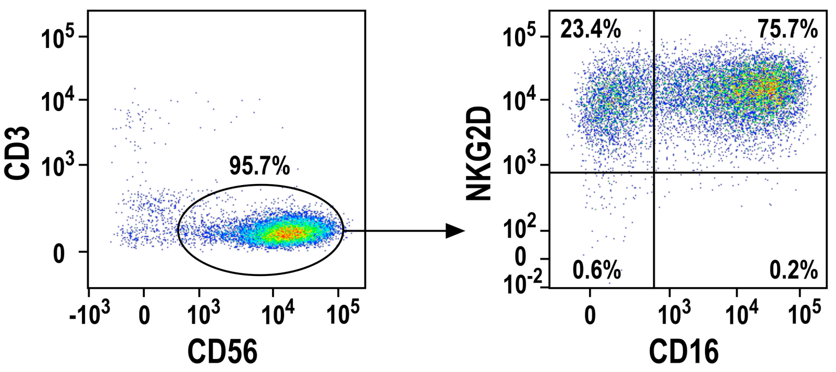

Figure S4. Purity of *ex vivo* expanded peripheral blood NK cells. pNK cells from healthy donors were isolated from buffy coats, and expanded *ex vivo* by stimulation with IL-2 and IL-15 for 3 days. Purity of the obtained cell population was analyzed by flow cytometry with CD3- and CD56-specific antibodies. In addition, NKG2D and CD16 expression by the gated CD56^{+} CD3^{-} NK cell population was assessed. Representative data from one donor are shown.

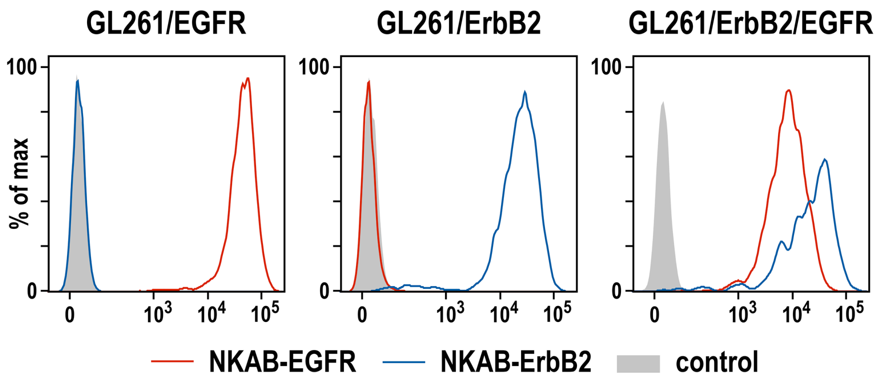

Figure S5. Binding of purified NKAB-EGFR (red lines) and NKAB-ErbB2 antibodies (blue lines) to GL261 glioblastoma cells expressing EGFR, ErbB2 or both target antigens was investigated by flow cytometry as indicated. Cells only incubated with secondary antibody (gray areas) served as controls.

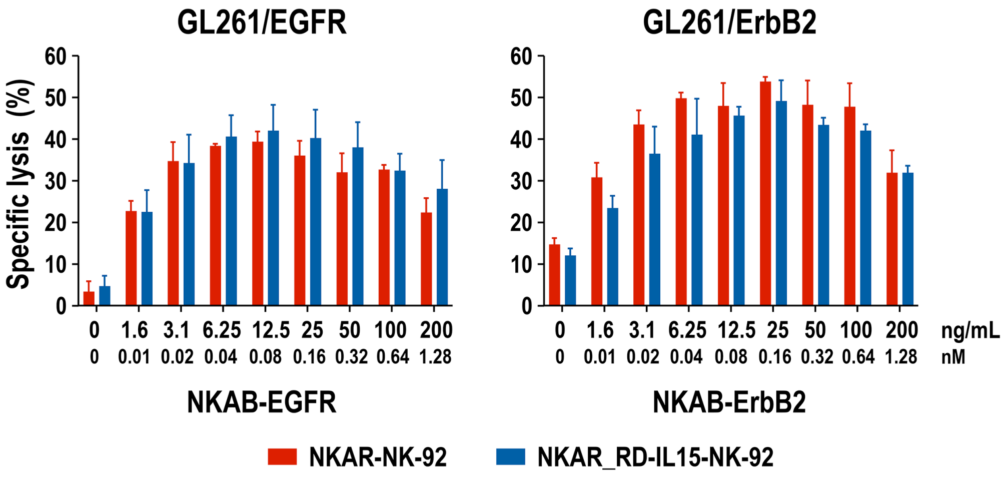

Figure S6. Lysis of GL261/EGFR (left) and GL261/ErbB2 cells (right) by NKAR-NK-92 (red bars) and NKAR_RD-IL15-NK-92 cells (blue bars) after 3 hours of co-culture at an E/T ratio of 5:1 in the absence or presence of increasing concentrations of NKAB-EGFR or NKAB-ErbB2 was investigated in a flow cytometry-based cytotoxicity assay. Mean values ± SD are shown; n=3 independent experiments.

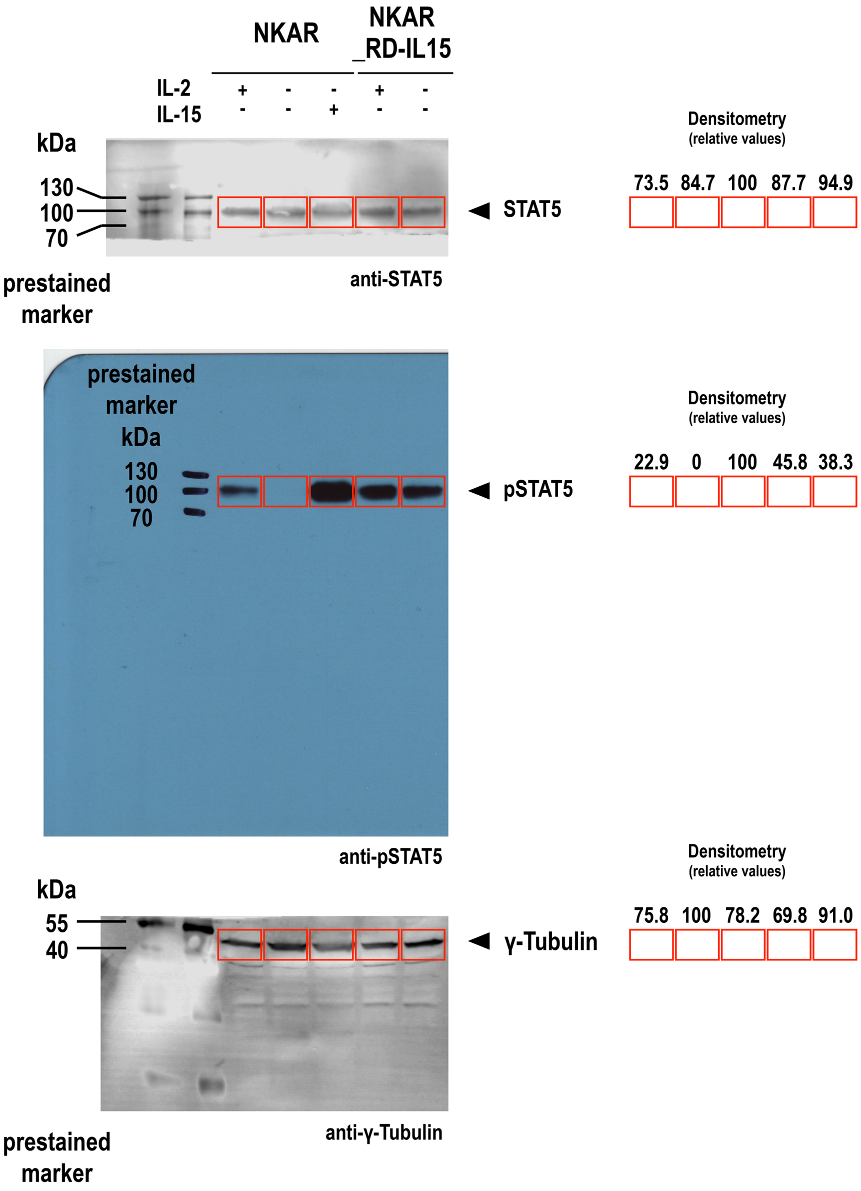

Figure S7. Uncropped images of the immunoblots shown in Figure 3B. Signals of respective protein bands were quantified using ImageJ version 1.54 software (National Institutes of Health, Bethesda, MD, USA; imagej.org). Values relative to the strongest signal in each blot are indicated.

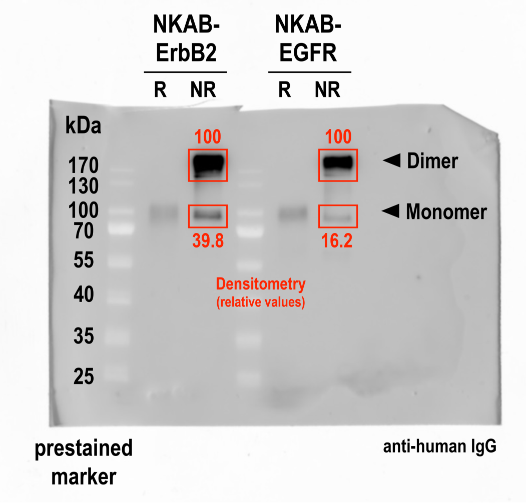

Figure S8. Uncropped image of the immunoblot shown in Figure 4B. Signals of protein bands representing dimers and monomers of NKAB-ErbB2 and NKAB-EGFR analyzed under non-reducing conditions (NR) were quantified using ImageJ version 1.54 software (National Institutes of Health, Bethesda, MD, USA; imagej.org). Values given for monomers are relative to the signals for the corresponding dimers as indicated.
